# Hypokalaemia and bradycardia unmask the loss-of-function phenotype of a Brugada Syndrome *SCN5A* mutation

**DOI:** 10.1093/europace/euaf160

**Published:** 2025-07-31

**Authors:** Anthony Frosio, Procolo Marchese, Giorgia Bertoli, David Molla, Martina Arici, Chiara Bartolucci, Chiara Piantoni, Giulia Guidi, Claudia Bazzini, Patrizia Benzoni, Raffaella Milanesi, Antonio Fortunato, Pierfrancesco Grossi, Luigi Pianese, Yi Wang, Riccardo Cappato, Marco Nardini, Stefano Severi, Annalisa Bucchi, Marcella Rocchetti, Mirko Baruscotti

**Affiliations:** Department of Biosciences, MiLab and “Centro Interuniversitario di Medicina Molecolare e Biofisica Applicata”, Università degli Studi di Milano, Milano 20133, Italy; Cardiology Unit, AST Ascoli Piceno, Ascoli Piceno, Italy; Department of Biosciences, MiLab and “Centro Interuniversitario di Medicina Molecolare e Biofisica Applicata”, Università degli Studi di Milano, Milano 20133, Italy; Department of Biosciences, MiLab and “Centro Interuniversitario di Medicina Molecolare e Biofisica Applicata”, Università degli Studi di Milano, Milano 20133, Italy; Department of Biotechnology and Biosciences, Università degli Studi di Milano Bicocca, Milan, Italy; Department of Electrical, Electronic and Information Engineering Guglielmo Marconi, University of Bologna, Cesena, Italy; Department of Biosciences, MiLab and “Centro Interuniversitario di Medicina Molecolare e Biofisica Applicata”, Università degli Studi di Milano, Milano 20133, Italy; Department of Electrical, Electronic and Information Engineering Guglielmo Marconi, University of Bologna, Cesena, Italy; Department of Biosciences, MiLab and “Centro Interuniversitario di Medicina Molecolare e Biofisica Applicata”, Università degli Studi di Milano, Milano 20133, Italy; Department of Biosciences, MiLab and “Centro Interuniversitario di Medicina Molecolare e Biofisica Applicata”, Università degli Studi di Milano, Milano 20133, Italy; Department of Biosciences, MiLab and “Centro Interuniversitario di Medicina Molecolare e Biofisica Applicata”, Università degli Studi di Milano, Milano 20133, Italy; U.O.C. Clinical Pathology Unit, AST Ascoli Piceno, Ascoli Piceno, Italy; Cardiology Unit, AST Ascoli Piceno, Ascoli Piceno, Italy; U.O.C. Clinical Pathology Unit, AST Ascoli Piceno, Ascoli Piceno, Italy; College of Pharmaceutical Sciences, Zhejiang University, Hangzhou, China; Arrhythmia and Electrophysiology Department, IRCCS MultiMedica, Milan, Italy; Department of Biosciences, MiLab and “Centro Interuniversitario di Medicina Molecolare e Biofisica Applicata”, Università degli Studi di Milano, Milano 20133, Italy; Department of Electrical, Electronic and Information Engineering Guglielmo Marconi, University of Bologna, Cesena, Italy; Department of Biosciences, MiLab and “Centro Interuniversitario di Medicina Molecolare e Biofisica Applicata”, Università degli Studi di Milano, Milano 20133, Italy; Department of Biotechnology and Biosciences, Università degli Studi di Milano Bicocca, Milan, Italy; Department of Biosciences, MiLab and “Centro Interuniversitario di Medicina Molecolare e Biofisica Applicata”, Università degli Studi di Milano, Milano 20133, Italy

**Keywords:** Brugada syndrome, SCN5A, Hypokalaemia, Arrhythmias

## Abstract

**Aims:**

Loss-of-function (LOF) mutations of the cardiac Na^+^ channel (*SCN5A*) are causatively associated with the Brugada Syndrome (BrS). However, the onset of Ventricular Fibrillation (VF) is a rare event, and critical factors favouring the pathological phenotype remain often elusive. This study explores how concomitant triggering conditions may impact on VF onset in a symptomatic proband carrying the S805L/*SCN5A* BrS mutation.

**Methods and results:**

Clinical, in-vitro, numerical, and structural analyses were performed. A 67-year-old male was resuscitated after cardiac arrest, and clinical analysis upon hospitalisation revealed severe hypokalaemia (2.5 mEq/L). The ECG showed a coved type-I BrS pattern and the *SCN5A* mutation (S805L) was identified. Patch-clamp studies carried out in a heterologous expression system (HEK293 cells) revealed that WT/S805L channels exhibit two different phenotypes (normal and LOF); the main parameter controlling this distribution is the cell membrane potential. A protected/normal behaviour was observed at −80 mV; conversely, LOF occurred at more negative potentials (−100/−120 mV). Further analyses in isolated outflow tract ventricular cardiomyocytes showed that hypokalaemia (and bradycardia) induced diastolic potential hyperpolarisation, thus favouring the Na^+^ current LOF. Computational and molecular modelling confirmed our findings and revealed the structural determinant of this alteration.

**Conclusion:**

WT/S805L Na^+^ channels exhibit either a LOF or a wild-type-like behaviour depending on the membrane potential. Since hypokalaemia and slow pacing rate induce cell hyperpolarisation and the associated LOF, they represent concurrent elements creating the scenario responsible for the VF and cardiac arrest. These results may represent an interpretative paradigm applicable to other BrS mutations.

## Graphical Abstract

**Graphical Abstract:**
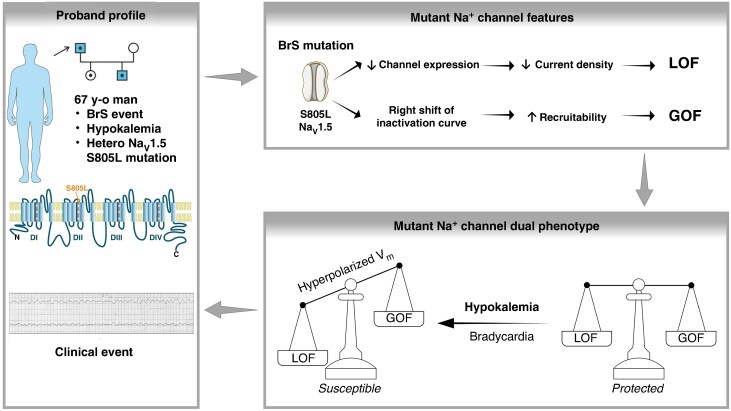

## Introduction

Brugada Syndrome (BrS) is an arrhythmic disorder characterized by conduction abnormalities that can result in ventricular fibrillation (VF) and sudden cardiac death.^1–3^ Affected individuals may manifest clinical events during adulthood or remain asymptomatic throughout their lives^4,5^. The severity of clinical manifestations has prompted robust efforts along two lines of interventions. The first is intended to identify individuals with spontaneous ≥2 mV ST elevation and negative T wave in V1 and V2 Electrocardiogram (ECG) leads at high risk of arrhythmic events with the aim of providing protective therapies, while the second is focused on the identification of the underlying molecular mechanisms. While genetic predisposition is well established^2,6–8^ dys-functional cardiac Na^+^ channels (Na_v_1.5) represent the only recognized mechanism supporting autosomal dominant inheritance of the disease^4,9^. Familial genetic co-segregation studies have identified Na_v_1.5 mutations in BrS patients, and *in-vitro* cellular and molecular analyses demonstrated that a reduction in the Na^+^ current (loss-of-function, LOF) is the functional signature of these mutant channels^4,10^ It is this LOF that is responsible for the so-called reduced cardiac conduction reserve in the Right Ventricular Outflow Tract (RVOT).^10,11^ However, the clinical manifestation of these mutations is far more complex since individuals carrying the same mutation may present with variable symptoms ranging from none to extreme severity, and males are at higher risk of being symptomatic^6,12–14^ Based on the evidence that the LOF is a lifetime genetically determined condition, while symptomatology is variable, it is concluded that the genetic *per se* cannot account for the complexity of the disease, and concurrent precipitating events must contribute to unleashing the pathological cascade.

In this study we present evidence that the functional manifestation of a *SCN5A* mutation (S805L), identified in family members affected by BrS, depends on the membrane potential. While at depolarized voltages heteromeric (WT/S805L) and wild-type (WT/WT) channels behave similarly, at more negative voltages WT/S805L channels display a LOF pattern. This dual behaviour is determined by two opposing and coexisting features: (i) a reduced channel protein expression (associated with a LOF) and (ii) an increased propensity to channel opening (gain-of-function, GOF). K^+^ serum oscillations directly influence the cell membrane potential and this dynamic balance, thus favouring the conditions for the pathogenic effect of the mutation in hypokalemic condition. Taken together, these aspects may explain why in normokalaemia the asymptomatic state is favoured, whereas hypokalaemia represents an arrhythmogenic condition favouring the manifestation of the Brugada event.

## Methods

### Genetic screening

A genetic screening for the identification of disease-causing genetic variants was carried out on a set of 174 genes associated with arrhythmias, and two variants of uncertain significance were reported: the p.Ser805Leu (S805L) mutation (caused by the substitution NM_198056.3:c.2414C > T) in the *SCN5A* gene (OMIM *600163) coding for the Na_v_1.5 cardiac Na^+^ channel (see Supplementary material online, *Figure S1*), and the p.Lys977Arg (K977R) mutation (caused by the substitution NM_001035.3:c.2930A > G) in the *RYR2* gene (OMIM *180902) coding for the cardiac ryanodine receptor type 2.

### Mutagenesis and heterologous expression

#### Mutagenesis

The S805L mutation (Serine TCG > Leucine TTG) was introduced into the genetic sequence of WT *SCN5A* (reference sequence NM_000335) through a commercial site-directed mutagenesis kit (*QuikChange II XL Site-Directed Mutagenesis Kit*, Agilent Technologies #200521), using the following primers:

F 5′ -TGTCCCGCATGAGCAACTTGTTGGTGCTGCGCTCCTTC-3′,

R 5′ -GAAGGAGCGCAGCACCAACAAGTTGCTCATGCGGGACA-3′.

Automated DNA sequence analysis (BioFab Research, Italy) was employed to verify the presence of the mutation in the channel sequence.

#### HEK293 cell culture and transfection

Human Embryonic Kidney 293 cells (HEK293, Sigma-Aldrich, #CB85120602; #RRID CVCL_0045) were used as a heterologous expression system for the evaluation of the biophysical properties of Na_V_1.5 WT and S805L mutant channels in the presence of the Na^+^ channel β1 subunit and the enhanced green fluorescent protein (EGFP) reporter. Cells were cultured in Dulbecco’s Modified Eagle’s Medium (DMEM, Life Technologies, #11965-0*92) supplemented with 10% Fetal Bovine Serum (FBS, Life Technologies, #10270098, lot 42Q5260 K), 1% Na^+^ pyruvate (Sigma-Aldrich, #S8636), 1% L-Glutamine (Sigma-Aldrich, #G7513), 0.5% Penicillin and 0.5% Streptomycin (0.1 mg/mL and 100 U/mL, respectively—Sigma-Aldrich, #P4458) at a controlled temperature of 37°C and 5% CO_2_.

The ViaFect™ transfection reagent (Promega, #E4981) was used to transiently transfect HEK293 cells with pCI Mammalian Expression Vector (GenBank U47119.2) containing either the WT-*SCN5A* (kindly provided by Dr. Flavien Charpentier—University of Nantes, France) or the mutated S805L-*SCN5A* and an additional pcDNA3.1 vector (GenBank OR659020.1) containing both the *SCN1B* cDNA sequence (NM_001037.4) and the *EGFP* reporter (kindly provided by Dr. Ilaria Rivolta—University of Milano Bicocca, Italy). 1 μg of WT-*SCN5A* was employed for the wild-type (WT/WT) condition, 1 μg of S805L-*SCN5A* for the Homo (S805L/S805L) condition, 0.5 μg of WT-*SCN5A*, and 0.5 μg of S805L-*SCN5A* for the Hetero (WT/S805L) condition. For every condition, 1 μg of *SCN1B*-*EGFP* was co-transfected. 48–72 h after transfection, cells were dispersed by trypsinisation and plated at low density on 35-mm plastic Petri dishes in the DMEM-based medium. Right before patch-clamp experiments the medium was replaced by standard Tyrode’s solution containing (mM): NaCl 140, KCl 5.4, CaCl_2_ 1.8, MgCl_2_ 1.0, D-glucose 5.5, and Hepes 5, pH 7.4 (300 mOsmol/L). Only EGFP-expressing cells were selected for the experiments.

### Animal procedures and cell isolation

All animal procedures performed in this study were carried out in accordance with the guidelines of the care and use of laboratory animals established by the Italian and UE laws (D. Lgs n° 2014/26, 2010/63/UE); the experimental protocols were approved by the Animal Welfare Committee of the University of Milano Bicocca and by the Italian Ministry of Health (protocol 29C09.N.YRR approved in June 2018).

#### Guinea-pig ventricular myocytes isolation

Female Dunkin-Hartley guinea pigs (175–200 g) were anesthetized by i.p. injection of xylazine (7.5 mg/kg) plus ketamine (130 mg/kg) and heparin (400 UI) provided by the University Committee for Animal Care and euthanized by cervical dislocation. Guinea-pig ventricular cells were then isolated by retrograde coronary perfusion with minor modifications.^15^ Ventricular myocytes from the right ventricular apex (RVA) and outflow tract (RVOT) were isolated for comparisons. Rod-shaped, Ca^2+^-tolerant myocytes were used within 12 h from dissociation. The guinea-pig model was chosen because of its similarity with human ventricular action potentials (APs).

### Electrophysiology

Electrophysiological data recordings: pCLAMP 11.1 (Molecular Devices, LLC). Data analysis and statistics: pCLAMP 11.1 (Molecular Devices, LLC); OriginPro 2021 (OriginLab Corp.); GraphPad Prims 5 (GraphPad Software Inc.); Graphical display: CorelDRAW X8 (Corel Corp.).

#### HEK293 cells

Na^+^ currents were recorded at room temperature in a low-Na^+^ external solution containing (mM): N-methyl-D-glucamine chloride 100, NaCl 30, CsCl 5, Hepes 10, MgCl_2_ 1.2, CaCl_2_ 2, and Glucose 5, pH 7.4, (278 mOsmol/L). Pipettes were pulled from borosilicate glass capillaries with resistance of 1–4 MΩ and filled with (mM): CsCl 130, NaCl 10, MgCl_2_ 1, Hepes 10, EGTA 10, ATP (disodium-salt) 2, pH 7.2 (298 mOsmol/L). On-line capacitance correction and series resistance compensation (40–80%) were employed. Current traces were low-pass filtered at a frequency of 10 kHz and sampled at a rate of 50 kHz. Na^+^ currents were elicited applying depolarising steps (40 ms duration, stimulation frequency 0.5 Hz) from −80 mV to +10 mV (increment 10 mV), while the cells were maintained at different holding potentials (HPs: −120, −100, −80 mV). To analyze the current density, the peak I_Na_ amplitude at each test potential was normalized to cell capacitance. Activation curves were then calculated and the experimental datapoints were interpolated by the Boltzmann distribution *y* = 1/(1 + exp(−(V−V½)/*s*)). Inactivation curves were obtained by eliciting the current at the fixed potential of 0 mV (50 ms duration, stimulation frequency of 0.625 Hz, HP −100 mV) after maintaining the cell at variable values comprised between −140 and −40 mV (1 s duration, 10 mV increment). The Boltzmann distribution *y* = 1/(1 + exp((V−V½)/*s*)) was used to interpolate experimental datapoints. Maximal conductance values (g_max_) were obtained by fitting g/V curves using the following equation g(V) = g_max_/(1 + exp(−(V−V½)/*s*)). In all equations V is the voltage applied, V_½_ is the half activation (or inactivation) voltage and *s* is the inverse of the slope factor.

#### Ventricular cardiomyocytes

During measurements myocytes were superfused (2 mL/min) at 35.5°C with Tyrode's solution containing (in mM): 154 NaCl, 2 CaCl_2_, 1 MgCl_2_, 5 HEPES/NaOH and 5.5 D-glucose, pH 7.35 (315 mOsmol/L); extracellular [K^+^] was controlled by adding KCl (2.5 mM, 3.5 mM or 5 mM) as required. Borosilicate pipettes (1.5–2 MΩ) were filled with (mM): 130 K^+^-aspartate, 10 NaCl, 2 CaCl_2_, 2 MgCl_2_, 10 HEPES-NaOH, 5 EGTA-KOH, 0.1 GTP (disodium salt), 2 ATP (disodium salt), and 5 creatine phosphate (disodium salt), pH 7.2 (297 mOsmol/L).

Acetylcholine (ACh, 10 µM)-induced K^+^ current (I_K(Ach)_) was elicited in V-clamped RVOT or RVA myocytes at −40 mV; its effects on AP parameters (AP duration at 90% of repolarisation -APD_90_- and diastolic membrane potential -E_diast_) were evaluated in I-clamp mode (10 kHz sampling rate and 2 kHz filter) during pacing at 1 Hz. In both cases, cells were superfused with Tyrode’s solution containing 3.5 mM KCl.

### Western blotting

Transfected HEK293 cells were lysed in 100 µL of RIPA buffer (Sigma Aldrich, # R0278) in the presence of a protease inhibitor cocktail (Sigma Aldrich, # P8340) and then snap-frozen in liquid nitrogen. After gently shaking for 10 min at 4°C, the lysate was spun at 4500*×g* for 5 min, and the supernatant was saved and stored at −80°C until use. Protein concentration was quantified by means of a Bicinchoninic Acid Assay (BCA, Sigma Aldrich, #SLBS8667). The protein extracts were then heated at 99°C for 5 min in sodium dodecyl sulfate-polyacrylamide gel electrophoresis (SDS-PAGE) solubilising buffer (58 mM Tris HCl, 10% glycerol, 2% SDS, 0.004% bromophenol blue, pH 6.8) containing 2.5% β-mercaptoethanol (Life-Technologies, #31350010). The proteins (5 µg/lane) were separated by means of SDS-PAGE on a 4–12% polyacrylamide gel (NuPAGE™ Bis-Tris, Life Technologies, #NW04120 Box) and transferred to a polyvinylidene difluoride membrane (PVDF, Bio-Rad, # 1620177). Blotted proteins were exposed to tris-buffered saline with Tween 20 (TBST) blocking solution (mM): Tris HCl 20, NaCl 150, pH 7.5, Tween20 0.1% plus 5% milk powder (Blotting Grade Blocker Non-Fat Dry Milk; Bio-Rad, #170-6404), and the membrane was then incubated overnight at 4°C with the anti-Na_v_1.5 (Sigma-Aldrich; dilution 1:250, #S1946) or the anti-actin primary antibodies (Sigma Aldrich, dilution 1:3000, #A3853) diluted in the blocking buffer. After several washes in TBST, the membrane was then incubated with the secondary antibodies (dilution in blocking buffer 1:10000) conjugated to horseradish peroxidase (Bio-Rad, # 1706515 and 1721011). The Pierce Pico ECL system (Life Technologies, # 32106) was used for detection. After each experiment, the PVDF membrane was always stained using the amido black (Bio-Rad, #161-0402) staining procedure to assess the efficiency of protein transfer and verify equal loading. The bands were densitometrically analyzed using the ImageJ software.

### Numerical simulations

Two different mathematical reconstructions of human ventricular AP were used. The most recent Bartolucci-Passini-Severi (BPS) model,^16^ which was specifically developed to correctly reproduce AP changes upon variations in extracellular electrolyte concentrations, and the well-established O’Hara-Rudy (ORd) model.^17^ The epicardial cell type was chosen since BrS-related arrhythmias are found in the epicardial zone of the RVOT.

To reproduce the S805L heteromeric (Hetero, WT/S805L) condition and compare it with the WT/WT one, the experimental parameters affected by the mutation have been modified in the models. In particular, to mimic the 26.9% decrease (from 2.64 to 1.93 nS) in the maximal conductance due to the mutation, the corresponding parameter for the fast component of the I_Na_ was reduced by the same factor. Regarding voltage-dependent kinetics, we set half-activation and inverse slope factors at the same experimental detected values.

To investigate the role of the beating rate we included in both models the extracellular cleft space based on Nygren *et al*.^18^ and Di Francesco and Noble^19^ works, in which they show the existence of local rate-dependent K^+^ accumulation in the extracellular space near the membrane. The cleft volume was set at 13% of the total volume and the diffusion time constant was equal to 35 s.

The models were implemented in Matlab (Mathworks Inc., Natick, MA, USA) with a variable order solver (ode 15 s). The stimulus had an amplitude of −75 µA/µF and a duration of 1 ms. The simulation was run for 1000 beats to consider results in steady-state conditions. The duration of AP upstroke (AP_ud_) was quantified as the time to peak from the beginning of the pacing pulse to the AP upstroke.^20^ Structural analysis and superimposition were made by using the program Coot.^21^

### Statistical analysis

All data are presented as mean ± SEM values. Comparisons of the Na_v_1.5 current/voltage (I/V) curves were carried out at the peak of the distribution (−20 mV) by means of the one-way ANOVA followed by post-hoc Fisher test. Conductance, activation and inactivation curves were compared using the extra sum-of-squares *F* test. Western blot protein expression levels and the I_K(ACh)_ densities (RVOT vs. RVA) were compared by means of the two-sample *t*-test. Comparisons of the APD_90_ and E_diast_ modulation by ACh were done using the Student’s paired *t*-test. Comparisons of the rate- and of the extracellular K^+^ concentration (K_out_)-dependency of E_diast_ were done using the Two-Way Repeated Measurements ANOVA test; rate-induced changes in overall curve steepness were defined according to significance of the interaction. Computational and statistical analysis were carried out with OriginPro 2020 (OriginLab, Northampton, MA) and GraphPad Prism 5 (GraphPad Software, San Diego, CA). Statistical significance is indicated by *P*-values < 0.05. Normal distribution of the data was verified by the Shapiro-Wilk test. Sequence alignments were done using the GeneDoc software.

## Results

### Clinical history and genetic investigation

**Figure 1:**
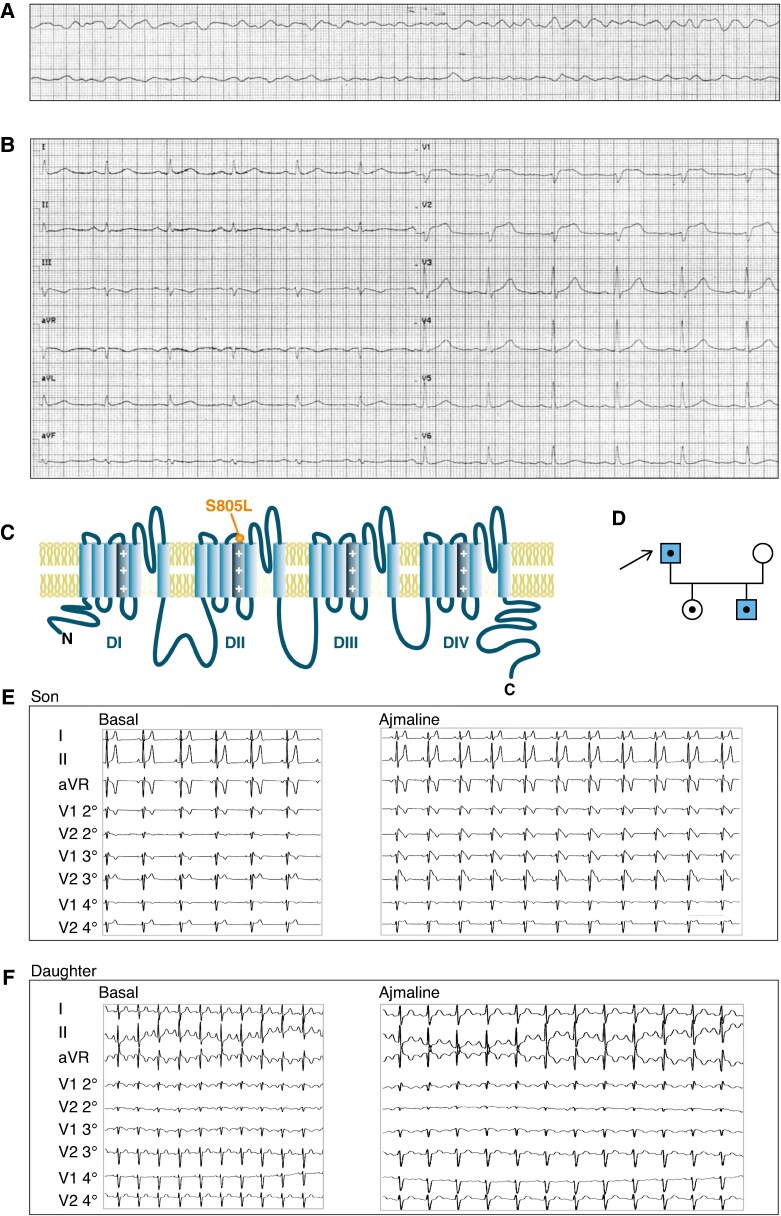
ECG phenotypes and familial inheritance of the S805L mutation. (*A*) ECG of the proband collected during cardiac arrest. (*B*) Type 1 BrS ECG pattern of the proband at hospital admission after resuscitation from cardiac arrest. (*C*) Schematic topology of the hNa_v_1.5 channel; the S805L mutation is located at the extracellular limit of the DIIS4 TM segment. (*D*) Family tree of the proband (arrow); coloured background indicates the presence of BrS pattern in the ECG; black dots indicate the presence of the heterozygous S805L mutation. (*E, F*) Ajmaline test (1 mg/Kg in 5 min) was positive in the son and negative in the daughter.

A 67 y-o man without previous medical history was referred to the intensive care unit for a previous cardiac arrest (*Figure 1A*). At hospital admission the patient complained of mild asthenia and dizziness, and the Glasgow Coma Scale score was 15. Primary vital signs (body temperature, blood pressure, heart rate, respiratory rate, and oxygen saturation) were normal and blood tests showed significant hypokalaemia (2.5 mEq/L) which was later corrected. His admission 12-leads ECG showed a significant BrS Type 1 pattern: J-point elevation with in leads V1 and V2 (with a small ST-segment notch in lead V1) without any reciprocal ST depression in complementary leads (*Figure 1B*). Structural heart disease and coronary artery disease were excluded by echocardiography and angiography. In the previous 2 days the proband experienced viral gastroenteritis, fever (the first day), nausea, vomiting, and diarrhoea. Based on these clinical features, the patient underwent implantable cardioverter-defibrillator implantation. For comparison a follow-up ECG recorded 1 month after hospital discharge is presented in Supplementary material online, *Figure S2*; a sinus rhythm with type 1 Brugada pattern in lead V1 was confirmed. Of note a prolonged QTc (>440 ms in men) was detected (see Supplementary material online, *Table S1*).

The genetic screening identified the p.Ser805Leu (S805L) variant in the *SCN5A* gene coding for the Na_v_1.5 cardiac channel and the p.Lys977Arg (K977R) variant in the *RYR2* gene coding for the cardiac ryanodine receptor type 2 (details in Methods). Interestingly, the S805L variant was previously associated with the BrS,^22^ but its functional significance was not explored at cellular level. Since the cardiac Na^+^ channel (Na_v_1.5) is currently recognized as the sole protein causatively associated with the BrS*^9^*, we focused on the Na_v_1.5 Ser805Leu mutation (*Figure 1C*, Supplementary material online, *Figure S1*) to uncover possible links between the cardiac disease and its underlying molecular mechanisms.

The proband had neither medical nor familial history of BrS, but the heterozygous Na_v_1.5 S805L mutation was identified both in his 29 y-o son and 34 y-o daughter (*Figure 1D* and Supplementary material online, *Figure S1C*, *D*). Ajmaline provocation test (1 mg/Kg in 5 min) induced type I BrS pattern in the son but not in the daughter (*Figure 1E, F*).

The S805 residue is evolutionary conserved both in several human Na_v_ and Ca_v_ channel isoforms and in cardiac Na_v_1.5 across the animal kingdom (see Supplementary material online, *Figure S1B*, *E*, *F*) and this stability implies functional relevance.

### Dual behaviour of mutant S805L channels: wild-type-like and loss-of-function

**Figure 2:**
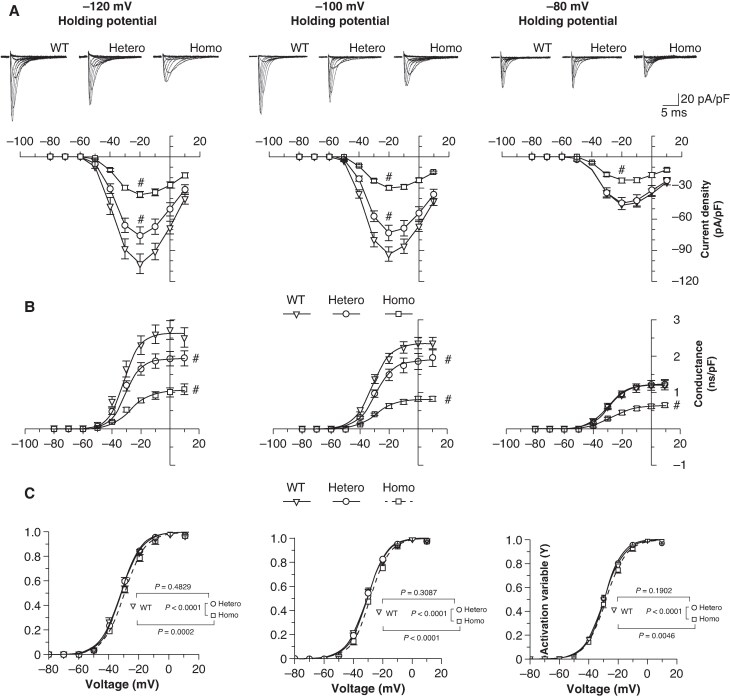
The electrophysiological behaviour of the heterozygous S805L mutation depends on the cell HP. Current/voltage (I/V, *A*) and conductance/voltage (g/V, *B*), and activation (*C*) curves obtained using HPs of −120 (left), −100 (middle), and −80 (right) mV in HEK293 cells transfected with WT only (WT, ▽), WT and S805L (Hetero, ○) and S805L only (Homo, □) channel constructs. Sample currents (40 ms duration, range: −80 to +10 mV) are shown at the top of panel A. g/V curves were fitted by the Boltzmann equation (g(V)=g_max_/(1 + exp(−(V−V½)/*s*))). Statistics were carried out on the peak of the I/V distribution (−20 mV) and on the g_max_ values. Peak currents, *n*, and statistical *P* values are provided in Supplementary material online, *Table S2*; g_max_, *n*, and statistical *P* values are provided in Supplementary material online, *Table S3*. Statistic test: one-way ANOVA followed by post-hoc Fisher test; ^#^  *P* < 0.05 vs. WT. Activation curves were fitted by the Boltzmann equation (*y* = 1/(1 + exp(−(V−V½)/*s*))). V½ and *s* values are provided in Supplementary material online, *Table S4*. Statistical curve comparisons (*P* values in the insets) were carried out using the Extra sum of squares *F* test.

We next *in-vitro* analyzed the current-voltage (I/V) and conductance-voltage (g/V) relations by expressing homomeric (Homo, S805L/S805L), heteromeric (Hetero, WT/S805L), and wild-type (WT, WT/WT) channels in a cell (HEK293) model system (*Figure 2A, B*). In literature these studies are usually performed by eliciting the current from very negative holding potential (HP ≤ −120 mV) to ensure maximal channel recruitability (fraction of channels that can open upon an incoming stimulus, i.e. the opposite of refractoriness). Since this is not the membrane potential normally met by cardiac channels, we chose to repeat the experiments also at more depolarized HPs (−100 mV and −80 mV) to better mimic the physio-pathological conditions of cardiac cells (*Figure 2*).

Analyses of current amplitudes and conductances shown in *Figure 2A, B* demonstrate that the Homo I_Na_ was consistently reduced in comparison to the WT at all HPs (see Supplementary material online, *Tables S2* and *S3*). The Hetero I_Na_ condition was however, differently affected by the HPs, since both the peak of the I/V and the g_max_ values were reduced at HPs −100 mV (−21.5% and −18.8%, respectively) and −120 mV (−26.4% and −26.9%, respectively), but no changes were detected at HP −80 mV. We next analyzed the voltage dependence of activation at the three HPs (*Figure 2C* and Supplementary material online, *Table S4*), and statistical comparisons did not reveal significant differences between Hetero and WT, thus a pathological relevance was excluded. We also explored possible mutation-induced effects on the persistent I_Na_ measured as TTX-sensitive current at −20 mV and we found no evidence of an impact of the S805L mutation on this aspect (see Supplementary material online, *Figure S3*).

**Figure 3:**
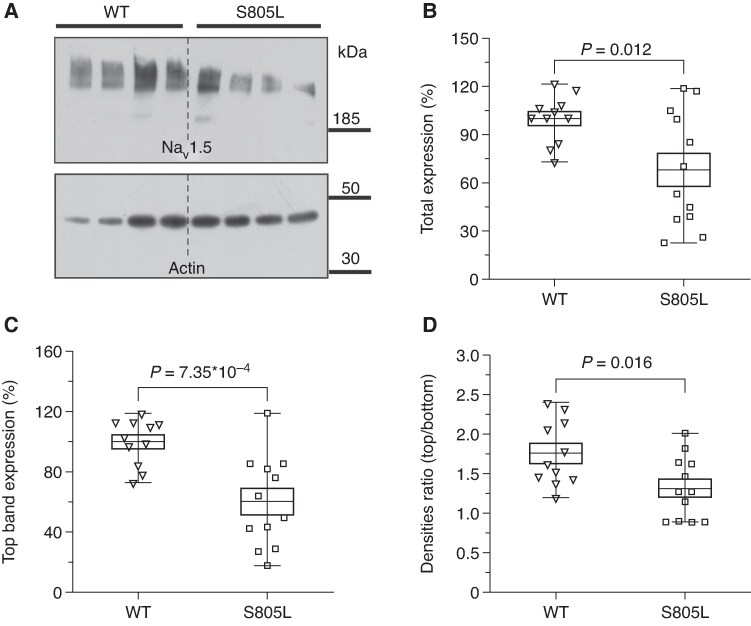
Western blot analysis of S805L homo and WT channel expression in HEK293-transfected cells. (*A*) Representative blot of total protein extract of HEK293 cells expressing WT and S805L channels. Each lane was loaded with 5 µg of total protein extract obtained from an independent culture dish; top and bottom bands likely represent two different glycosylation states of the channel. (*B, C*) Densitometric analyses of 3 independent experiments of top + bottom (total, B) and top (*C*) bands; S805L signals were decreased (vs. WT) by 31.9% and by 40.2% (*n* = 11, 12), respectively. Data were normalized to endogenous actin and expressed as % of WT values. (*D*) Ratio of the top vs. bottom channel forms; for the S805L Homo channel the ratio is decreased by 25.1% (*n* = 11, 12). Box plot: middle line, mean value; extremities, SEM; whiskers, maximum and minimum values. Statistics: two-sample *t*-test.

While the differences observed using a HP −120 mV (i.e. under maximal recruitability) point to a reduced protein/channel expression (loss-of-expression LOE) for the Hetero and Homo conditions, the lack of effect observed for Hetero channels at HP −80 mV required an additional and different explanation. The reduced expression of S805L mutant protein was also evaluated by means of Western Blot experiments; indeed, a double band signal (*Figure 3A*), likely reflecting the fully (top band) and partially glycosylated (bottom band) channel forms was detected.^23^ Densitometric analyses confirmed that the total (top + bottom bands) expression of S805L channels was reduced by 31.9% (*Figure 3B*), and the decrease is further exacerbated when the analysis is restricted to the top band (−40.2%, *Figure 3C*). The top/bottom band ratio was also evaluated both for WT and for S805L channels and a 25.0% decrease was observed (*Figure 3D*).

**Figure 4:**
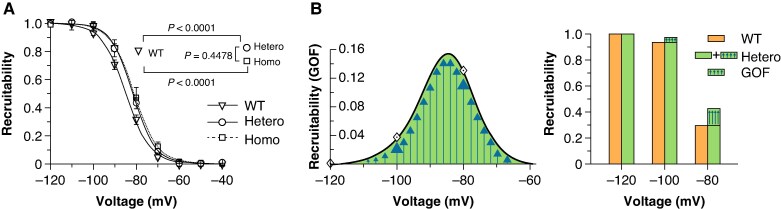
The S805L mutation induces a GOF of the voltage-dependent inactivation. (*A*) Experimental data and Boltzmann fitting of mean fractional inactivation values for the WT (*n* = 34 cells), Hetero (*n* = 20), and Homo (*n* = 12) conditions, respectively. V_½_ and *s* values are presented in Supplementary material online, *Table S4*; Homo and Hetero curves are significantly different from the WT (statistics are shown in the inset). The Extra sum-of-squares *F* test was used for statistical comparison. (*B*) The bell-shaped curve (left panel) corresponds to the difference between the Hetero and the WT inactivation curves shown in panel A and represents the GOF acquired by mutant channels. Diamonds represent the GOF factors at the holding value of −80, −100 (indicated by large arrows), and −120 mV. The bar-graph (right panel) illustrates the recruitability of WT (orange) and Hetero (green). The presence of the arrows in the green area indicates the GOF of Hetero channels.

The reduced expression of S805L channels well explains the LOF behaviour observed with HPs −120 and −100 mV; however, it is not compatible with the evidence that at the more depolarized HP (−80 mV) WT and Hetero channels behave similarly (*Figure 2*). This apparent inconsistency was investigated by analyzing the voltage dependence of inactivation of the WT, Homo, and Hetero channels since this parameter quantifies the fraction of channels that can open upon an incoming pacing stimulus (recruitability). This analysis revealed that the recruitability of the Hetero and Homo conditions was positively shifted compared with the WT (*Figure 4A* and Supplementary material online, *Table S4*), indicating that at physiological potentials (−100/−80 mV) a larger fraction of the Hetero (vs. WT) channels can be recruited for opening; this increase thus represents a gain-of-function (GOF).

This GOF was quantified and illustrated by plotting the difference between the Hetero and WT recruitability curves (the bell-shaped curve of *Figure 4B*, left). The GOF associated with the Hetero condition at voltages of −80 mV (+13.0%) and −100 mV (+3.7%) are better visualized in the bar-graph of *Figure 4B*, right. This voltage-dependent increased availability (GOF) is therefore at work to counteract the decrease in channel expression (LOE).

Data presented so far show that, for Heteromeric WT/S805L channels, the LOF prevails at HP −120/−100 mV (*Figure 2A, B*), while at −80 mV the contribution of the GOF (*Figure 4*) becomes relevant with a balanced GOF/LOF impact so that WT and Hetero currents are similar. On the other hand, for Homo S805L/S805L channels, the LOF largely prevails at all HPs (*Figure 2*). We therefore speculated that physiological conditions favouring cell hyperpolarisation, hence less Na^+^ current, could set the stage for the BrS manifestation in the Hetero conditions.

### Hypokalaemia and slow rate favour cell hyperpolarisation and the LOF manifestation

Given the established connection between vagal predominance and sudden cardiac death episodes^4,24^ we first evaluated whether the ACh-induced current (I_K(ACh)_) was expressed in cells isolated from the guinea-pig RVOT (site of emergence of the BrS;^10,25^) for comparison we also analyzed cells of the RVA. Data shown in Supplementary material online, *Figure S4* confirm the presence of the I_K(ACh)_ in both cell types, but larger in RVOT cells. Accordingly, ACh shortened APD_90_ in both cell types (RVA: −10%, RVOT: −14%), without significantly affecting diastolic potential (E_diast_). This ruled out vagal activity as a possible direct cause of ventricular cell hyperpolarisation. We therefore explored the possibility that other important factors such as hypokalaemia and rate of stimulation could be involved in the hyperpolarizing process.

**Figure 5:**
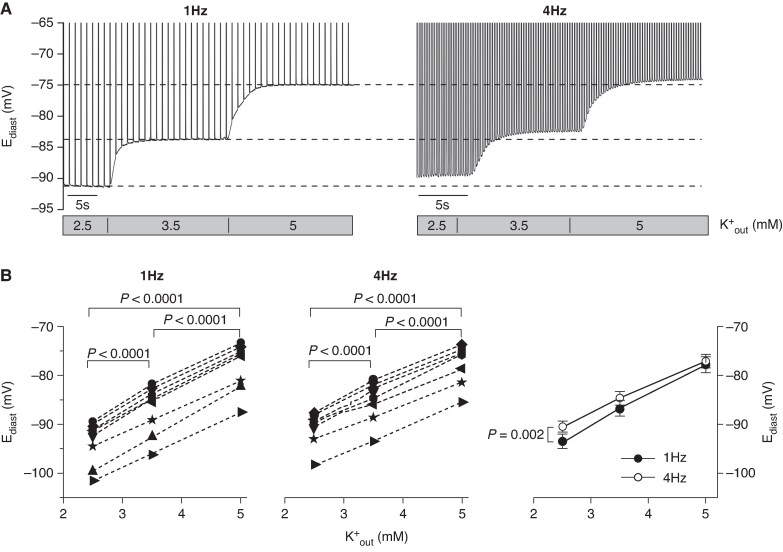
Hypokalaemia and stimulation rate modulate the diastolic potential (E_diast_) of RVOT cardiomyocytes. (*A*) Sample APs recorded in a cardiomyocyte stimulated at 1 (left) and 4 (right) Hz, and sequentially exposed to 2.5, 3.5, and 5.0 mM extracellular K^+^ concentrations (K ^+^  _out_). The top part (>−65 mV) of the AP traces has been removed for clarity. Dashed lines correspond to steady-state E_diast_ levels recorded at 1 Hz. (*B*) Single E_diast_ values measured in *n* = 9 RVOT myocytes stimulated at 1 (left) and 4 (right) Hz, and sequentially exposed to 2.5, 3.5, and 5.0 mM extracellular K^+^ concentrations (as in panel A); mean ± SEM E_diast_ (see also Supplementary material online, *Table S5*) are presented on the right panel. Left and middle panels, statistics: RM one-way ANOVA followed by post-hoc Fisher test. Right panel, statistics: RM Two-way ANOVA; internal comparison reveals a significant interaction between K^+^_out_ and rate.

Comparative experiments were carried out in guinea-pig RVOT cells (*Figure 5*) using three extracellular K^+^ concentrations (K^+^_out_, 2.5, 3.5, 5 mM) and two stimulation rates mimicking *in-vivo* bradycardic and normal rate conditions (1, 4 Hz). The 2.5 mM K^+^_out_ matched the hypokalemic condition of the patient upon hospitalisation, while 3.5 and 5 mM K^+^_out_ correspond to the lower/upper limits of normal K^+^ serum levels.^26^

Sample APs recorded in a cell exposed to the different experimental conditions listed above are shown in *Figure 5A* (for graphical clarity AP points > −65 mV are not shown). Proper quantitative evaluation is presented in *Figure 5B* (original data in Supplementary material online, *Table S5*). Statistical comparison of the mean curves (*Figure 5B*, right) confirmed that the E_diast_ hyperpolarisation was significantly associated with the progressive hypokalemic condition both at 1 and 4 Hz and reveals a robust hyperpolarizing shift of 16.5 mV when the extreme conditions are considered (5 mM K^+^/4 Hz: E_diast_ = −77.0 ± 1.3 mV and 2.5 mM K^+^/1 Hz: E_diast_ = −93.5 ± 1.4 mV). Similar experiments repeated in cells isolated from the RVA confirmed the dependence of E_diast_ on K^+^_out_ and rate (see Supplementary material online, *Figure S5* and *Table S6*).

### Action potential computational reconstruction

Given the potential clinical implication of this finding, we next assessed the impact of the mutation by inserting the experimental I_Na_ parameters into the BPS human ventricular AP computational model^16^ and by simulating hypokalemic and bradycardic conditions.

**Figure 6:**
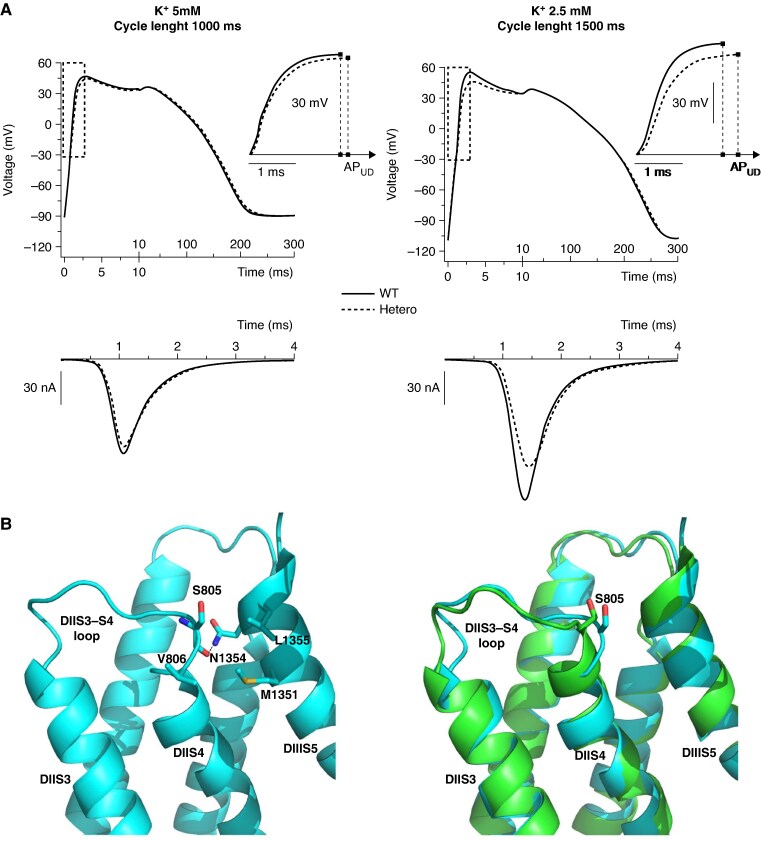
Human AP simulations and structural 3D prediction support the pathogenic role of the S805L mutation. (*A*) Computed human ventricular APs (top) and the corresponding WT and Hetero I_Na_ traces (bottom) simulated in basal (left: 1 Hz, 5 mM K^+^_out_) and hypokalemic and bradycardic (right: 0.66 Hz, 2.5 mM K^+^_out_) conditions. APs are presented using a dual time scale to better appreciate the different shapes; the areas identified by dotted rectangles are enlarged in the inset to better illustrate the difference in the time required to reach the peak of the upstroke phase (AP_ud_). (*B*, left) Position of the S805 residue in the DIIS3-S4 loop. Relevant residues at the interface between DIIS4 and DIIS5 are shown in stick representation and labelled. H-bond is shown as dashed a line. (*B*, right) Structure superimposition of voltage-gated Na^+^ channel Na_v_1.5 6LQA (green) and 7DTC (cyan).

In *Figure 6A* WT and Hetero human AP (top) and Na _v_1.5 current (bottom) simulations obtained in conditions of normal K^+^_out_ and rate (5 mM, 1 Hz, left) and of hypokalaemia and bradycardia (2.5 mM, 0.66 Hz, right) are shown. The simulation carried out in hypokalemic and bradycardic conditions yielded an E_diast_ ∼20 mV more negative than that in physiological condition (see Supplementary material online, *Table S7*) and revealed that, in comparison to the WT condition, the Hetero AP has a reduced upstroke (−8.7 mV) and a slower depolarisation rate. Alteration of the RVOT depolarisation rate is considered an important element associated with the BrS^4,27^ For this reason, we used the AP upstroke duration parameter (AP_ud_, i.e. time to upstroke, *Figure 6A top* and insets) to quantify this alteration, and we observed an increase (vs. WT) of the Hetero AP_ud_ by 7.4% in 5 mM K^+^/1 Hz and by 13.8% in 2.5 mM K^+^/0.66 Hz (see Supplementary material online, *Table S7*).

The impact of bradycardia and hypokalaemia were also evaluated independently to ascertain their relative contribution to the combined effects on AP parameters: hypokalaemia alone was by large the most important contributor, while the effect of bradycardia was marginal (see Supplementary material online, *Table S7*). Similar results (see Supplementary material online, *Figure S6* and *Table S7*) were obtained using a different computational model (O’Hara-Rudy model).^17^

### Structural interpretation of S805L mutation

The S805 residue is located in a loop (DIIS3-S4) at the extracellular boundary of the DII-S4 transmembrane helix (*Figure 1C*). Cryo-EM structures of human Na_v_1.5 channels (PDB-codes: 6LQA, 7DTC) show that the S805 contributes to the anchoring of the DII-S4 helix to the DIII-S5 helix by H-bonding N1354 (*Figure 6B*, left). Furthermore, the DIIS3-S4 appears to be flexible, assuming more than one conformation in the cryo-EM structures (*Figure 6B*, right) and, therefore, the S805L mutation (polar vs. hydrophobic side chain) is likely to promote a reorientation of the L805 toward the nearby hydrophobic pocket lined by V806, M1351, and L1355 (*Figure 6B*, left), thus partially reshaping the DIIS3-S4 region. Structural heterogeneity of the DIIS3–S4 region is also evident in other two recently solved Nav1.5 α-subunit structures (PDB: 8VYJ, 8VYK), where nearby cholesteryl hemisuccinate induces conformational changes in the DIIS3–S4 region affecting DIII–S5 interactions.^28^ In these structures, V806 replaces S805, fitting into a hydrophobic cleft formed by M1351 and L1355, supporting the hypothesis that the pathological S805L mutation induces a similar rearrangement of the DIIS3-S4 region.

## Discussion

BrS is an open clinical and scientific challenge due to its elusive nature and often deadly outcome. BrS as well as other channelopathies affect only a small proportion of cardiac patients; however, they provide valuable study models to identify the molecular alterations causatively associated with the integrated concept of ‘pathological state’. Indeed, the evolution in knowledge on the genetic basis of arrhythmias harbours the seeds of precision and patient-tailored medicine.^29^ Prevention and therapy will therefore largely benefit from advancement in mechanisms elucidation and identification of triggering conditions such as those presented in this study.

The genetic screening of the *SCN5A* gene identified the putatively pathogenic S805L mutation in the proband and in his two children (*Figure 1C, D*); of note, the S805 residue is conserved among several human isoforms and across evolution (see Supplementary material online, *Figure S1*). In line with the established prevalence of BrS in males,^12,13^ the ajmaline provocative test unmasked a type-I BrS pattern in the ECG of the son but not of the daughter (*Figure 1E, F*). Brugada phenotype is indeed sex dependent with a male prevalence of about 8–10 times; also, men are more likely to have pathological ECG, more symptoms, and a greater chance of inducible ventricular arrhythmias during electrophysiology studies.^30–32^ Given the autosomal nature of Brugada mutations, it is reasonable that sex-related modifiers may favour the clinical phenotype in males or have a protective influence in females.^33^ Interestingly, testosterone serum levels impact on the degree of right precordial ST-segment amplitude (a phenomenon that is reversible after orchidectomy and androgen deprivation.^34,35^) It is therefore conceivable that, despite the S805L mutant genotype, the Ajmaline challenge in the proband’s daughter was not enough to elicit a disease phenotype because of sex-related modifiers.

The presence of the type-I ECG pattern at time of admission is indicative of a possible BrS and the presence of S805L mutation in the patient and in family members corroborates the diagnosis. The association of this mutation with the BrS is in line with a previous similar finding.^22^

The literature on cardiac BrS Na_v_1.5 mutations shows that a LOF is full-bodied.^23,36–38^ A reduced Na_v_1.5 current is a condition shared by both the repolarisation and depolarisation hypotheses associated with the alterations observed in the BrS.^10^ Our S805L study demonstrates that this paradigm holds, but it is subtler than expected. Indeed, heteromeric channels functionally switch from a WT-like to a LOF phenotype according to the cell membrane potential (*Figure 2A, B*) and this switch depends on a dynamic equilibrium between the reduced expression of Na_v_1.5 mutant proteins (LOE, *Figure 3*) and the GOF caused by a different voltage-dependent recruitability (*Figure 4*). At physiological membrane potential values, the two effects tend to null each other, and the Hetero and WT currents are similar; however, as the membrane potential becomes progressively more negative (*Figures 2* and *4*), the GOF is reduced. Therefore, any event that favours a marked hyperpolarisation would unleash the LOF phenotype exposing individuals carrying the mutation to a higher risk of BrS manifestation. The association between resting membrane potential hyperpolarisation and hypokalaemia has been reported both at the single-cell level and in intact cardiac tissue.^39–41^ Sicouri and Antzelevitch^40^ report a 10.8 mV hyperpolarisation of M cells when halving the K^+^_out_ (4 to 2 mM) and this value is compatible with the 9.9–15.6 mV hyperpolarisation range observed in our experiments (K^+^_out_: 5 to 2.5 mM, data from Supplementary material online, *Tables S5* and *S6*). The association between single-cell and intact tissue data is therefore quantitative sound, supporting the transferability of the cellular findings to the clinical observation.

A decrease in Na_v_1.5 peak current is considered predictive of the clinical presentation and penetrance of the BrS^42^; however, in line with our findings, there is ample evidence that Na_v_1.5 mutations cannot always fully account for the occurrence of the clinical manifestation, and additional triggering factors such as vagal predominance, bradycardia, electrolyte imbalances, hyperthermia, and Na^+^ channel blocking drugs are important comorbidities*^4^*. We therefore searched for factors affecting the LOF/GOF balance, and the experiments presented in *Figure 5* and Supplementary material online, *Figures S4*–*S6*, explored the physiological stimuli that could lead to cell hyperpolarisation. Vagal alteration and the associated sinus bradycardia are recognized elements associated with BrS^4,10^; however, despite the presence of the cholinergic modulation of the APD in RVOT and RVA cells, no ACh-dependent hyperpolarisation of E_diast_ was observed (see Supplementary material online, *Figure S4*) excluding this mechanism as a S805L-dependent BrS trigger.

A large E_diast_ hyperpolarisation was instead present both in RVOT and RVA cells when external K^+^ was lowered from physiological levels (3.5–5 mM^43^) to clinically relevant hypokalaemia (2.5 mM; *Figure 5* and Supplementary material online, *Figure S5* and *Tables S5*, *S6*). Since the E_diast_ hyperpolarisation reduced the GOF (*Figure 4*), a coherent mechanism supporting the dynamic balance between a ‘protected’ (depolarized) and a ‘susceptible’ (hyperpolarized) state emerges. Indeed, while the association between hypokalaemia and an increased risk of ventricular arrhythmias is an established concept,^44^ recent findings suggest an association also with the BrS. For example, hypokalaemia-induced lethal events were described in 2 BrS patients^45^ and evidence that correcting the hypokalemic conditions (1.6–2.9 mM) of patients may resolve the BrS ECG pattern is also reported.^46,47^ Similarly, prior to the BrS event, the proband of our study experienced a marked hypokalemic condition (K^+^ 2.5 mEq/L) induced by vomit and diarrhoea. Besides hypokalaemia, we also considered bradycardia as a potential trigger of the BrS. Lowering the stimulation rate of RVOT cells from 4 to 1 Hz caused an additional E_diast_ hyperpolarisation (*Figure 5*), but, by itself, this hyperpolarisation appears too small to be causative. Serum K^+^ levels have been reported to oscillate according to a circadian rhythm with lower values prevalent from the late afternoon to the mid of the night,^48^ which reasonably corresponds to resting/bradycardic period and to the prevalent time-window of BrS manifestation.^30^ This observation suggests that the concomitant physiological nocturnal changes in kalemia and heart rate can contribute to the more susceptible arrhythmogenic substrate in BrS patients during night. Moreover, our data are in line with the observation that a low K^+^ ‘may unmask Type I Brugada ECG pattern’.^46^

When the experimental *in-vitro* data were used in computational reconstructions of the human ventricular AP, a decrease in the I_Na_ peak current and an increase in the AP_ud_ parameter were observed (*Figure 6A* and Supplementary material online, *Figure S6*). The evidence that the AP upstroke of a single ventricular myocyte is delayed by hundreds of µs is associated with a slower propagation of the AP from cell to cell.^20^ This cellular electrical derangement could possibly represent the functional substrate for the onset of clinical manifestations.

The S805 residue is evolutionary highly conserved among Na^+^ channel isoforms thus suggesting an important structural and functional role; of note, several voltage-dependent Ca^2+^ channels also exhibit a serine residue in the same structural position (see Supplementary material online, *Figure S1F*). Data shown in *Figure 6B* (right) suggest that the presence of the S805L mutation partly disturbs the spatial orientation of the DIIS3-S4 region and destabilizes the anchoring of the DII-S4 helix to DIII-S5 helix. It is this structural alteration that ultimately affects the voltage dependence of the channel and creates the molecular substrate for the Brugada phenotype. This structural interpretation is in line with recent observations associated with arrhythmias by Jiang *et al.*^36^ which concluded that the S3-S4 integrity is important to ensure proper gating function. Finally, it should also be considered that by removing the serine residue, the S805L mutation disrupts a glycosylation sequence (NLS) and this may decrease protein stability and introduce possible trafficking defects.

Overall, this study shows that under normal conditions, the balance between the GOF and LOF behaviours of the S805L mutation guarantees a ‘protected’ WT-like Na^+^ current phenotype. By favouring the hyperpolarisation of the E_diast_, hypokalaemia and bradycardia move the balance toward a LOF phenotype paving the way to the onset of the arrhythmic event.

### Study limitations

This study describes the dys-functional properties of a mutant channel identified in a Brugada patient and attempts to associate the LOF behaviour and the presence of additional hypokalemic conditions with the clinical onset of VF. A limitation of the study is that its conclusions apply only to the mutation (S805L) under investigation. However, since the Na^+^ current LOF feature is a hallmark of BrS genetics, it is conceivable that our interpretative framework might also apply to other BrS ion channel mutations. Unfortunately, a retrospective analysis of the literature is limited by the absence of specific experiments such as the ones described in this study.

A further caveat of the study is that the biophysical properties of the mutated channel were explored in a heterologous expression system (HEK293 cells). Patient-specific induced pluripotent stem cell-derived cardiomyocytes could also be considered as a model; however, given their relatively depolarized resting membrane potential, they may not be appropriate for this type of investigation.

## Supplementary Material

euaf160_Supplementary_Data

## Data and materials availability

All data are available upon request to corresponding authors.
