# Supplementary material for: Hypokalaemia and bradycardia unmask the loss-of-function phenotype of a Brugada Syndrome SCN5A mutation
Source: Europace. 2025 Jul 31;28(2):euaf160. doi: 10.1093/europace/euaf160 (PMC12886553; doi:10.1093/europace/euaf160)
Supplement: euaf160_Supplementary_Data [file euaf160_supplementary_data.pdf]

SUPPLEMENTARY MATERIAL

Hypokalemia and bradycardia unmask the loss-of-function phenotype of a Brugada Syndrome *SCN5A* mutation

Anthony Frosio[1,8#], Procolo Marchese[2#], Giorgia Bertoli[1,9#], David Molla[1], Martina Arici[3], Chiara Bartolucci[4], Chiara Piantoni[1,10|], Giulia Guidi[4], Claudia Bazzini[1], Patrizia Benzoni[1], Raffaella Milanesi[1,11], Antonio Fortunato[5], Pierfrancesco Grossi[2], Luigi Pianese[5], Wang Yi[6], Riccardo Cappato[7], Marco Nardini[1], Stefano Severi[4], Annalisa Bucchi[1], Marcella Rocchetti[3*], Mirko Baruscotti[1*].

#These authors contributed equally to this work

*Corresponding authors. Emails: mirko.baruscotti@unimi.it; marcella.rocchetti@unimib.it

INDEX

Supplementary Figures____ pagg. 2-7

Supplementary Tables ____ pagg. 8-10

References____ pag. 11

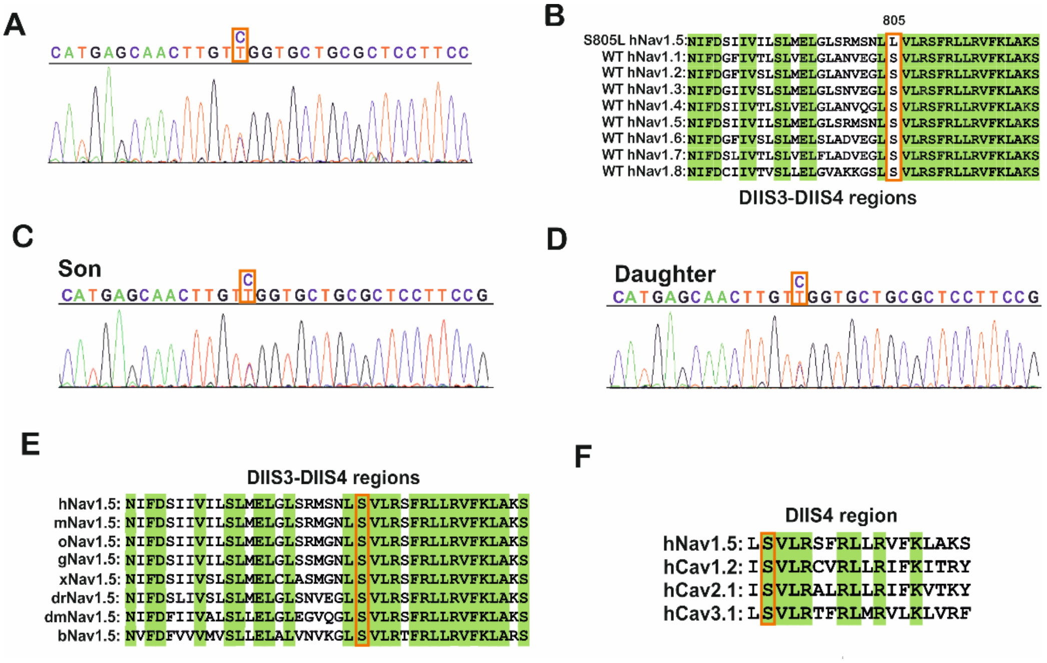

Supplementary Figure S1. Sequence analyses and comparative alignments of wild-type (S805) and mutant (S805L) residues. (**A**) Sequencing electropherogram showing the presence of the heterozygous c.2414C>T (p.S805L) mutation in the DNA of the patient. (**B**) Sequence alignment of the DIIS3-DIIS4 region of the $Na_v1.5$ S805L mutant and of several wild-type human Na^+ channel isoforms; the S805 residue shows a high level of conservation. (**C, D**) Electropherograms of the proband's son and daughter showing the presence of the mutation in both individuals. (**E**) Evolutionary conservation of the $Na_v1.5$ S805 residue; the S805 residues are in the orange box. (**F**) Sequence alignment of human voltage dependent Ca^{2+} channels and the $hNa_v1.5$; the S805 residue (orange box) is conserved (green). m, *Mus musculus*; o, *Oryctolagus cuniculus*; g, *Gallus gallus*; x, *Xenopous laevis*; dr, *Danio raerio*; dm, *Drosophila melanogaster*; b, *Branchiostoma laceolatum*.

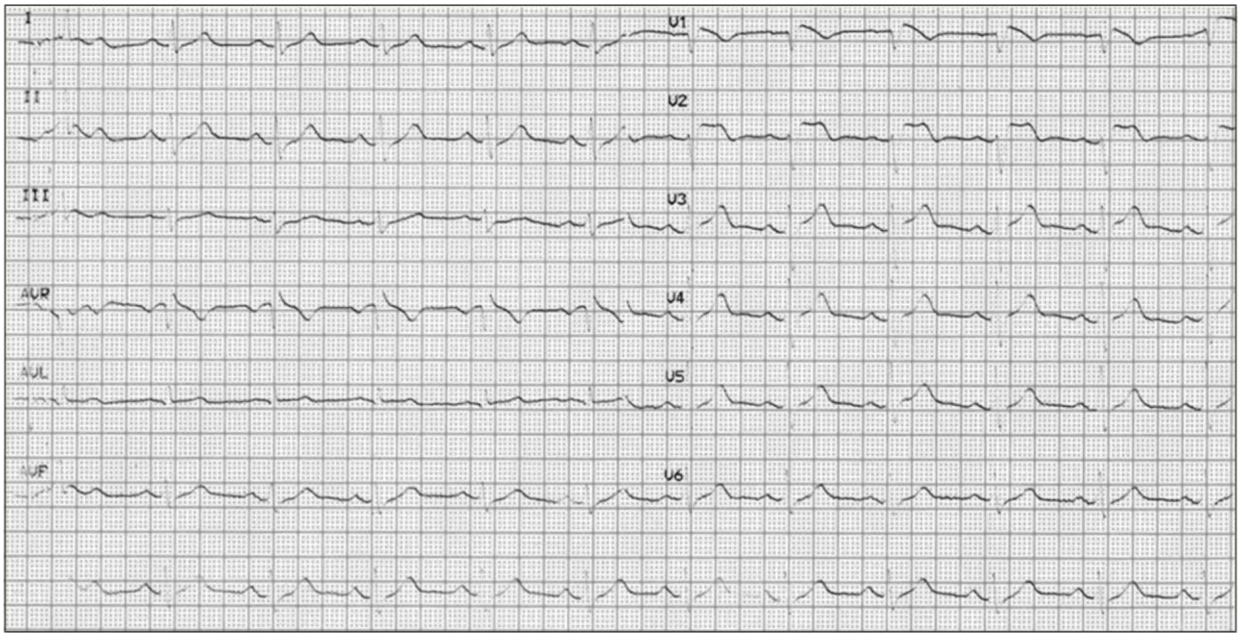

Supplementary Figure S2. ECG of the proband recorded 1 month after patient discharge. The 12 leads ECG with V1-V2 placed in parasternal 2°, 3°, and 4° intercostal spaces indicates coved type 1 Brugada pattern.

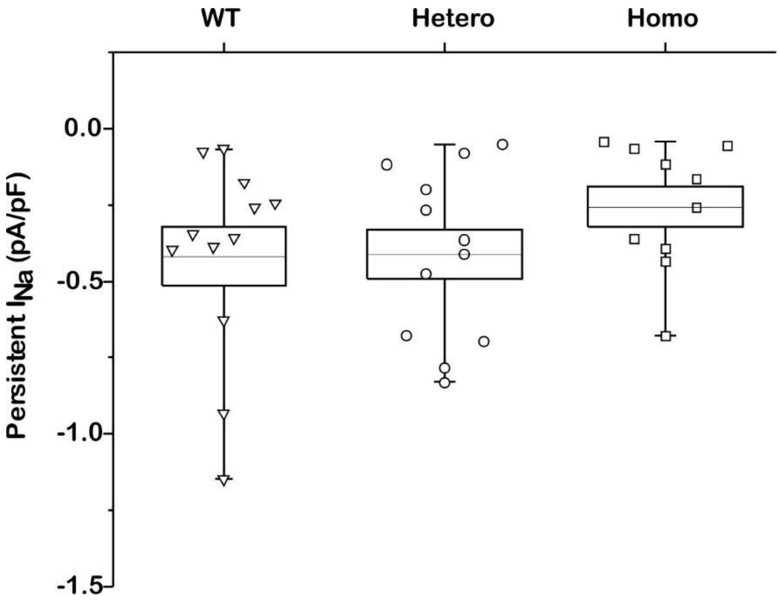

Supplementary Figure S3. Mean±SEM persistent Tetrodotoxin (TTX, 30 µM, Hello Bio, UK # HB1035)-sensitive I_{Na} densities measured at the end of a 220 ms long test pulse at -20 mV from a HP of -120 mV. WT: 0.42±0.10 pA/pF, n=12; Hetero: 0.41±0.08 pA/pF, n=12; Homo: 0.26±0.07 pA/pF, n=10.

Box plot: middle line, mean values; extremities, SEM; whiskers, maximum and minimum values.

Statistics: One Way ANOVA, P= 0.33986. Not significantly different.

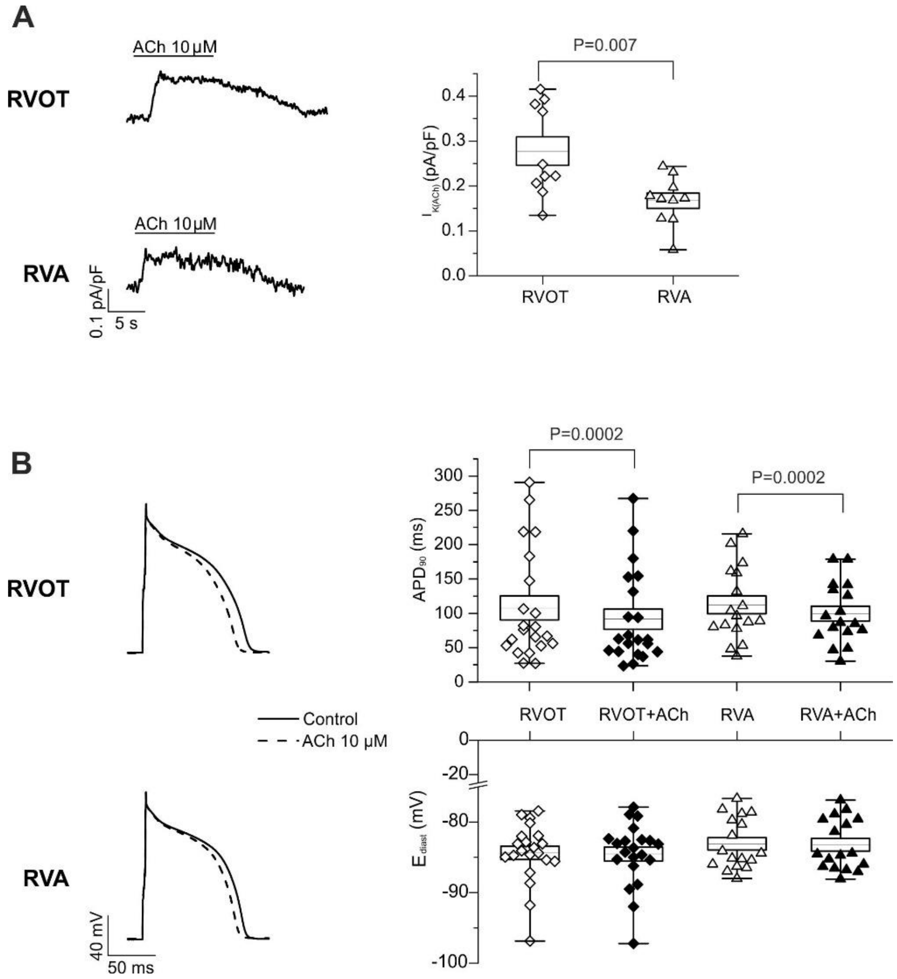

Supplementary Figure S4. ACh induces $I_{K(ACh)}$ but does not modulate E_{diast} in guinea-pig RVOT and RVA cardiomycytes. (**A**) Sample $I_{K(ACh)}$ current traces (left) elicited by ACh (10 µM) at HP=-40 mV in cells isolated from the guinea-pig right ventricular outflow tract (RVOT) and apex (RVA); mean peak current densities±SEM (right): RVOT, +0.28±0.03 pA/pF, n=10; RVA, +0.17±0.05 pA/pF, n=10. Statistics: Student's unpaired t-Test. (**B**) Sample stimulated AP traces (left) and plots of the AP duration at 90% of repolarization (APD_{90}) and diastolic membrane potential (E_{diast}) data distributions (right). Statistical comparison of these two parameters measured in the absence and in the presence of ACh (10 µM) revealed that ACh similarly shortened the APD_{90} by 10.5±1.3% and by 13.9±1.5% in the RVA (n=17) and RVOT (n=21), respectively, but failed to modify E_{diast}. Statistics: Student's paired t-test. Box plots: the middle line represents the mean value, the extremities the SEM, and the whiskers maximum and minimum values.

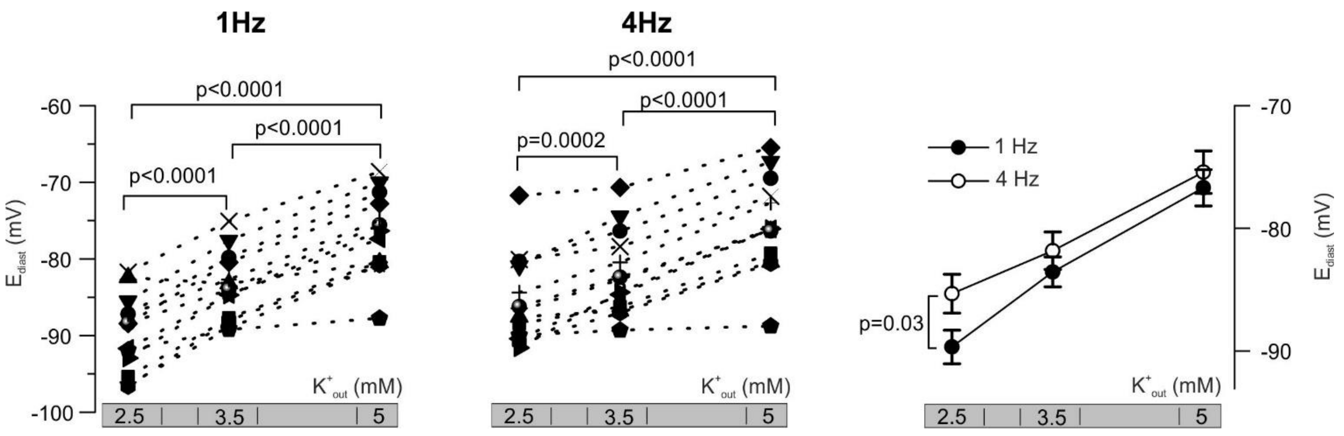

Supplementary Figure S5. E_{diast} values obtained in RVA cells exposed to different K^+_{out} concentration and stimulation rates. Single E_{diast} values measured in n=13 RVA myocytes during stimulation at 1 (left) and 4 Hz (middle), and sequentially exposed to 2.5, 3.5, and 5.0 mM extracellular K^+ concentrations (K^+_{out}). Mean±SEM E_{diast} values are shown in the right panel and reported in Supplementary data Table S6. Comparisons were carried out by RM one-way ANOVA followed by post-hoc Fisher test (left and middle) and by RM Two-way ANOVA (right).

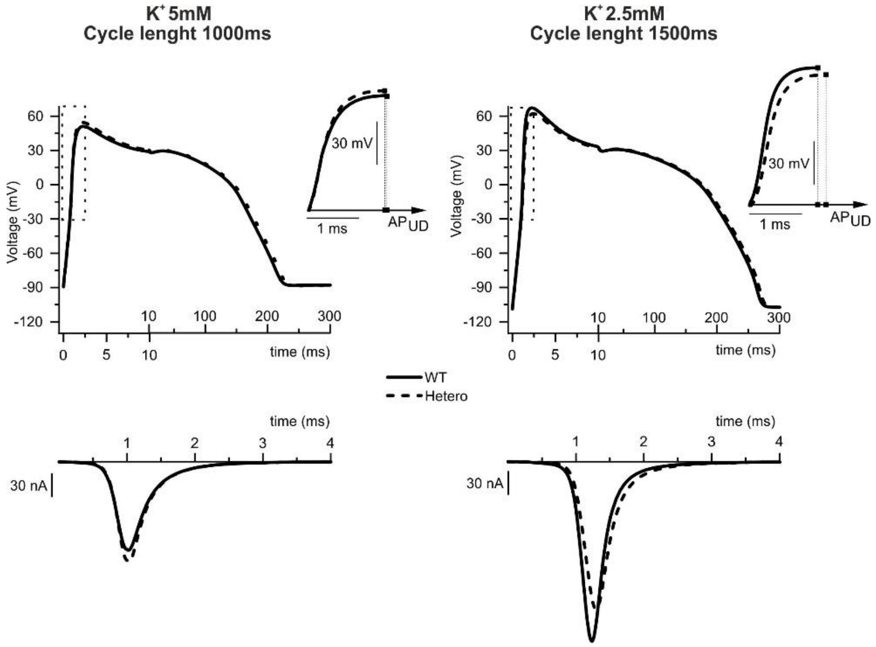

Supplementary Figure S6. Human action potential (AP) simulations with the O'Hara-Rudy (ORd) model[1]. Computed human ventricular APs (top) and the corresponding WT and Hetero I_{Na} traces (bottom) simulated in basal (left: 1 Hz, 5 mM K^{+}_{out}) and hypokalemic/bradycardic (right: 0.66 Hz, 2.5 mM K^{+}_{out}) conditions. APs are presented using a dual time scale to better appreciate the different shapes; the areas identified by dotted rectangles are enlarged in the inset to better illustrate the difference in the time required to reach the peak of the upstroke phase (AP_{ud}).

SUPPLEMENTARY TABLES

Supplementary Table S1. ECG parameters of the proband measured at hospital admission and 1 month after hospitalization.

	PR (ms)	QRS (ms)	QT (ms)	RR (ms)	QTc (ms)
**day 0 ECG** at hospital admission (*Figure 1B*)	180	120	430	880	460
**1 month follow up ECG** (*Figure S2*)	180	120	420	840	460

Supplementary Table S2. Peak I_{Na} amplitudes measured in heterologous expression system of WT and mutant channels.

	**I_{Na} peak @ -20 mV (pA/pF)**		
	**HP = -120 mV**	**HP = -100 mV**	**HP = -80 mV**
**WT**	-102.9±9.5 (n=15)	-93.0±6.8 (n=41)	-44.6±5.7 (n=20)
**Hetero**	-75.7±8.1 (n=24) P=0.0174	-73.0±7.5 (n=26) P=0.0238	-46.8±3.8 (n=26) P=0.7239
**Homo**	-36.2±3.1 (n=15) $P=1.565*10^{-6}$	-29.9±2.3 (n=33) $P=8.17*10^{-12}$	-22.6±2.5 (n=13) P=0.0034

Statistic comparison (vs WT) by one-way ANOVA followed by post-hoc Fisher test.

Supplementary Table S3. Maximal I_{Na} conductance (g_{max}) in heterologous expression system of WT and mutant channels.

	**g_{max} (nS/pF)**		
	**HP = -120 mV**	**HP = -100 mV**	**HP = -80 mV**
**WT**	2.64±0.18 (n=27)	2.34±0.12 (n=77)	1.19±0.08 (n=37)
**Hetero**	1.93±0.14 (n=47) P=0.001	1.90±0.16 (n=46) P=0.007	1.22±0.07 (n=47) P=0.70
**Homo**	1.05±0.10 (n=27) $P=1.44*10^{-9}$	0.81±0.05 (n=58) $P=1.20*10^{-18}$	0.64±0.4 (n=24) $P=1.08*10^{-5}$

Statistic comparison (vs WT) by one-way ANOVA followed by post-hoc Fisher test.

Supplementary Table S4. Boltzmann fitting parameters (V½ and s) of mean fractional activation and inactivation values.

	Fractional activation parameters					
	WT		Hetero		Homo	
	V½ (mV)	s (mV)	V½ (mV)	s (mV)	V½ (mV)	s (mV)
**HP = -120 mV**	-32.4 (n=15)	7.0	-30.9 (n=41)	6.8	-29.5 (n=19)	6.7
**HP = -100 mV**	-32.4 (n=24)	6.6	-30.7 (n=25)	6.4	-29.7 (n=28)	6.2
**HP = -80 mV**	-30.3 (n=15)	7.0	-28.6 (n=30)	6.8	-28.1 (n=13)	6.9
	**Fractional inactivation parameters**					
	**WT**		**Hetero**		**Homo**	
	**V½ (mV)**	**s (mV)**	**V½ (mV)**	**s (mV)**	**V½ (mV)**	**s (mV)**
	-84.9 (n=34)	5.7	-81.5 (n=20)	5.2	-80.9 (n= 12)	5.6

Statistic curves comparison were carried out using Extra sum of squares F test (P values are provided as insets of Figs. 2C and 4. Activation curves: at all potentials WT ≠ Homo and Hetero ≠ Homo; Inactivation curves: WT ≠ Homo and WT ≠ Hetero.

Supplementary Table S5. Diastolic membrane potentials (E_{diast}) measured at different external K^+ concentrations (K^+_{out}) and stimulation rates in Right Ventricular Outflow Tract (RVOT) cardiomyocytes (n=9).

K^+_{out}	E_{diast} 1 Hz	E_{diast} 4 Hz
**2.5 mM**	-93.49±1.43 mV	-90.53±1.10 mV
**3.5 mM**	-86.72±1.62 mV	-84.72±1.37 mV
**5 mM**	-77.84±1.57 mV	-77.04±1.34 mV

Statistic comparison by RM Two-way ANOVA reveals that rates of stimulation and K^+_{out} influence the E_{diast} value (P=0.02).

Supplementary Table S6. Diastolic membrane potentials (E_{diast}) measured at different external K^+ concentrations (K^+_{out}) and stimulation rates in Right Ventricular Apex (RVA) cardiomyocytes (n=13).

K^+_{out}	1 Hz	4 Hz
**2.5 mM**	-89.70±1.37 mV	-85.32±1.58 mV
**3.5 mM**	-83.55±1.23 mV	-81.82±1.53 mV
**5 mM**	-76.70±1.48 mV	-75.42±1.73 mV

Statistic comparison by RM Two-way ANOVA reveals that rates of stimulation and K^+_{out} influence the E_{diast} value (P=0.03).

Supplementary Table S7. Action Potential upstroke duration (AP_{ud}), maximal upstroke (UP_{max}), and diastolic membrane potential (E_{diast}) calculated using two ventricular AP computational models: Bartolucci-Passini-Severi (BPS model)[2] and O'Hara-Rudy (ORd model)[1].

		E_{diast} (mV)		UP_{max} (mV)		AP_{ud} (ms)	
		**BPS**	**ORd**	**BPS**	**ORd**	**BPS**	**ORd**
**1 Hz K^+_{out} = 5 mM**	WT	-88.8	-88.7	46.3	51.0	2.7	2.3
	Hetero	-89.4	-89.4	43.6	54.5	2.9	2.2
	Δ	-0.6	-0.7	-2.7	3.5	0.2	-0.1
**0.66 Hz K^+_{out} = 2.5 mM**	WT	-108.4	-108.7	54.2	67.1	2.9	2.3
	Hetero	-108.4	-108.8	45.5	62.1	3.3	2.5
	Δ	/	-0.1	-8.7	-5	0.3	0.2
**1 Hz K^+_{out} = 2.5 mM**	WT	-108.4	-108.6	53.2	65.4	3.0	2.2
	Hetero	-108.4	-108.5	44.6	60.5	3.3	2.4
	Δ	/	0.1	-8.6	-4.9	0.3	0.2
**0.66 Hz K^+_{out} = 5 mM**	WT	-88.7	-88.8	46.8	51.0	2.7	2.3
	Hetero	-89.3	-89.4	44.3	55.5	2.9	2.2
	Δ	-0.6	-0.6	-2.5	4.5	0.2	-0.1

REFERENCES

1. O'Hara T, Virág L, Varró A, Rudy Y. Simulation of the undiseased human cardiac ventricular action potential: model formulation and experimental validation. *PLoS Comput Biol*; 2011;**7**.
2. Bartolucci C, Passini E, Hyttinen J, Paci M, Severi S. Simulation of the Effects of Extracellular Calcium Changes Leads to a Novel Computational Model of Human Ventricular Action Potential with a Revised Calcium Handling. *Front Physiol*; 2020;**11,** art. 314.
